# Accounting for intimate partner violence perpetration. A cross‐cultural comparison of English and Brazilian male substance users' explanations

**DOI:** 10.1111/dar.12450

**Published:** 2016-08-12

**Authors:** Polly Radcliffe, Ana Flávia Pires Lucas d'Oliveira, Susan Lea, Wagner dos Santos Figueiredo, Gail Gilchrist

**Affiliations:** ^1^ National Addiction Centre, Institute of Psychiatry, Psychology and NeuroscienceKing's College LondonLondonUnited Kingdom; ^2^ Gender Violence and Health Research Group, Department of Preventive MedicineUniversity of São PauloSão PauloBrazil; ^3^ University of GreenwichOld Royal Naval CollegeLondonUnited Kingdom; ^4^ Centre for Biological and Health Sciences, Department of MedicineFederal University of São CarlosSão PauloBrazil

**Keywords:** intimate partner violence, perpetration, substance use, gender, account

## Abstract

**Introduction and Aims:**

This paper describes how substance use features in the accounts of intimate partner violence (IPV) perpetrators in treatment in England and Brazil. The aim of the research was to better understand cross cultural constructions of IPV perpetration amongst men in treatment for substance use.

**Design and Methods:**

Semi‐structured interviews were conducted with 40 men in community substance use treatment in Sao Paolo, Brazil and London and the South East of England who had reported IPV perpetration in a questionnaire survey. A thematic, narrative analysis was carried out of men's explanations for IPV perpetration.

**Findings:**

Three types of narratives were distinguished: (i) disputes, centred on substance use, that escalate to IPV perpetration; (ii) IPV perpetration that is explained by uncharacteristic loss of control, as a result of intoxication; and (iii) IPV perpetration provoked by a perceived betrayal, in which substance use is incidental. In all types of accounts hegemonic principles of male and female roles and behaviour provided a context for and make IPV perpetration explicable.

**Discussion and Conclusions:**

Substance use and IPV are culturally constructed and contextually defined. Understanding the meaning‐making of substance using IPV perpetrators has implications for the treatment of both substance abuse and IPV. [Radcliffe P, d'Oliveira AFPL, Lea S, dos Santos Figueiredo W, Gilchrist G. Accounting for intimate partner violence perpetration. A cross‐cultural comparison of English and Brazilian male substance users' explanations. *Drug Alcohol Rev* 2017;36:64–71]

## Introduction

Research suggests that intimate partner violence (IPV) perpetration is over‐represented in men in treatment for substance use 1, 2, 3. Surveys of IPV victims 4 and police reports 5 indicate that IPV offences are very often carried out when men are intoxicated with alcohol and other drugs. The relationship between drug and alcohol intoxication and IPV is never the less controversial 6, 7. Conceptions of IPV are contested 8, 9, 10, 11, 12 and there is little consensus regarding why some men who use alcohol and drugs are more likely to perpetrate IPV 8. Theories that examine how substance use interacts with culture, subculture, family and individual characteristics in IPV perpetration 13, 14 point to complex interactions.

Social scientists have long highlighted the way wrong doers account for their transgressions; almost 60 years ago Sykes and Matza 15 captured the verbal techniques whereby juvenile delinquents ‘neutralised’ their breaching of conventional values. Scott and Lyman 16 similarly described how offenders justified and excused transgressions. Narrative scholars 17, 18 argue that such practices are *constitutive* of social action; in other words, the narratives that give meaning to action also influence that action and make deviant behaviour possible. Maruna 19 argued that in order to desist from offending, the ex‐offenders he interviewed needed to construct coherent narratives of their criminal pasts that made sense of a new, reformed identity. In this paper we compare and analyse the narratives of men in drug and alcohol treatment who have been identified as IPV perpetrators in two contrasting national contexts, London and the South East of England and São Paulo, Brazil.

Alcohol intoxication in particular has been found to provide a common resource to explain and excuse IPV perpetration 20, 21, 22, 23, 24. Aside from the role of intoxication, however, little research has examined how substance use, IPV and gendered relations are linked in perpetrators' accounts and although it is known that both narratives 25, 26 and understandings of IPV 27 vary culturally, IPV perpetrators' accounts have not been considered cross‐culturally. This study seeks to enhance understandings of how substance use and normative conceptions of gender feature in IPV perpetrators' accounts in two contrasting cultural settings.

Rates of life time, non‐sexual domestic abuse victimisation reported by women in household surveys in Sao Paolo and England and Wales are intriguingly similar (at 27 and 22% respectively) 4, 28. Brazilian society is in transition and processes of re‐democratisation in the last 30 years have created space for feminist and women's activism that have resulted in significant moves towards building gender equality and away from the former patriarchal order 29. Women's police stations 30 where women can report crimes of sexual and physical violence and the Maria de Penha law 31, 32 provide measures to address IPV perpetration and victimisation. Challenges remain in the implementation of these policies however 33. In the UK, IPV remains a significant problem with estimates that two women are killed by a partner or ex‐partner per week 34. While there are sets of safeguarding procedures for victims of IPV, there are also high levels of attrition for reports of IPV offences 35 and research has revealed police decision‐making practices that systematically discount IPV reports as legitimate cases for prosecution 36. Thus as elsewhere, in neither England nor Brazil do, police and sentencers' responses and practices always reflect legislative intent 27.

## Methods

The qualitative research this paper is based on the second phase of a study investigating the prevalence of IPV amongst men receiving substance use treatment in São Paulo, Brazil and London and the South East of England 37. Convenience samples of 223 men in England and 281 men in São Paulo were recruited from treatment waiting rooms to take part in questionnaire interviews. Those who reported having perpetrated IPV (including physical, psychological and sexual violence) were informed after the questionnaire interview about the possibility of an additional in‐depth interview. Forty men (20 from London, small towns and one city in the South East of England and 20 from São Paulo) were purposively sampled to include a range of ages, types of substance and violence perpetrated in order to generate the maximum range of perspectives and experiences 38. This sample size was calculated to be large enough for comparison between locations and to achieve data saturation 39. Four men in English sites and two men in Brazilian sites who were eligible refused to take part in further research.

Of the 20 English research participants, 12 were recruited from two National Health Service community addiction services in south London. The other eight participants were recruited from voluntary sector, non‐government organisation substance misuse services in three towns in South East England: a formerly industrial town, a regional administrative centre (each with populations of over 100 000 inhabitants) and a coastal city. Although the participants from London were more ethnically diverse, the English research participants were remarkably homogenous in terms of their levels of education, social class and substance use. The Brazilian sample was recruited from five services, which like the English services were community based, out‐patient centres for alcohol and drug users [Centro de Atencao Psicossocial Alcool e Drogas (CAPS‐AD)] in São Paulo, Brazil. One was directly provided by the Brazilian health service, Sistema Único de Saúde, while the other four were contracted out by the health service to non‐government organisations. One of the services was located in a residential area near the University of São Paulo medical school. The other four out‐patient centres (CAPS‐AD) were located in working class areas with favelas and low budget houses on the periphery of São Paulo.

There are marked differences in substances used in Brazil and England that are reflected in our sample (Table 1). Whereas in the UK, heroin use, combined with crack cocaine and alcohol, dominates the profile of those in treatment for substance use 40, in Brazil there is very little heroin, but high use of crack cocaine and alcohol 41, 42. Both Brazilian and English research participants had high levels of comorbid hazardous drinking.

**Table 1:** Characteristics of the sample

	England	Brazil
*Age*		
Age range	33–58	29–59
Mean age	41.45	43
*Ethnicity*		
White	15	7
Black/mixed heritage/South Asian	5	13
*Substance use treatment*		
Heroin or heroin crack	13	0
Alcohol	3	10
Alcohol/crack	1	10
Abstinent	3	0
*Current relationship*		
Relationship	8	7
No relationship	12	13

The English interviews were conducted by two experienced, white, female researchers. The Brazilian interviews were conducted by five male and two female interviewers who received training in qualitative interviewing. Two of the Brazilian male interviewers were Black. English participants were given £20 (in cash or gift‐voucher depending on the services' preference) to compensate them for their time. Following local protocol, Brazilian research participants were not compensated to take part. Interviews took place in treatment service counselling rooms. All interviews were based on informed consent. Participants were assured that their responses would be anonymised and would not compromise access to future treatment and that confidentially would only be breached if they referred in the course of the interview to harming themselves or someone else. The interviews lasted between 45 and 90 min, were digitally recorded and transcribed verbatim. Informants were allocated pseudonyms. Ethical approval for this study was granted by the Comitê de Ética em Pesquisa da secretaria de Saúde da Prefeitura de São Paulo (Ref: 715.462) and East Midlands‐Northampton National Research Ethics Service in England (Ref: 14/EM/0088).

The interviews were designed to elicit men's accounts of perpetrating IPV and how IPV related to substance use, intoxication and a substance using lifestyle. Detailed questions were asked about relationships, conflicts and specific incidents of violence and how they had been resolved. A coding frame was developed by PR based on an initial set of themes derived from the interview schedule. This was shared and developed with GG and SL and with AF and WF at a project meeting in São Paulo. Three Brazilian interview transcripts were translated into English and each research team shared three transcripts. Data were discussed and further collaborative coding took place in subsequent Skype call meetings. The coding was then refined to themes 43. Because the focus of the research is men's conceptions of the role of substance use in IPV perpetration, the approach involved attention to the language used in men's accounts and to narrative 17. In illustrating our analysis with interview quotes we refer to men's anonymised names, substance used and whether they are from England or São Paulo (SP).

### Findings

In our analysis we discuss four main ways in which men talked about substance use, IPV and gendered relations. These are: (i) the impact of substance use on lives and relationships; (ii) substance use as the misspending of time and money; (iii) the direct impact of intoxication; and (iv) respect, sexual jealousy and violent control of women.

### The impact of substance use

Researchers have noted how the transformative power of drug and alcohol intoxication is commonly used to explain IPV 44, 45. Here we examine how men in our study referred to the effects of a transition into dependent drug use and how assuming a drug using lifestyle provided the context for stress and tensions that could escalate to violence:

*I was with Partner1, wasn't on drugs. I was completely clean. She didn't meet me on drugs. And then I started taking drugs and the person I became she didn't like*. (Keith, treatment for heroin, hazardous drinking, England).

*Drugs led me to the street, took me out of my own house, took me away from everything I had. I sold my car…I sold everything I have, like, of any worth. Drugs, what do they do? They anaesthetise, and they make you forget those good moments, right? Make you forget you have kids*. (Augusto, hazardous drinking, crack and cocaine, SP).

*When I was 25 I was earning £150 a day so money wasn't exactly a big issue because I had it. But then drugs take over, you stop going to work, you lose your job and then money is a big issue. And if you've got my drug habit, her drug habit and no money*. (Kenneth, treatment for heroin, hazardous drinking, England).

Becoming a dependent drug user is described by Keith as character changing. It is drugs, Augusto suggests that *made* him homeless and ‘took him away from everything [he] had’. Kenneth's statement that ‘drugs take over’ was an expression that was frequently used in interviews. Research participants thus present their experience of dependent substance use as transformative. They posit substance use—and the activities required to raise funds to purchase drugs—as both replacing all other meaningful activities and damaging to family relationships.

### Substance use as the misspending of time and money

The theme of arguments escalating to violence as a result of the misspending of time and money was common. Research participants described the *sneakiness* and unexplained absences involved in undisclosed substance use:

*She thought I was having an affair, but I was having an affair with drugs*. (Kevin, treatment for heroin and crack, England).

Men commonly described violence as a consequence of their absences from home in order to use substances on which they had spent limited household funds. Roni describes disputes over his entitlement to spend time and money he perceived belonged to him:

*So the reason for…for these discussions was really for me wanting to be right, wanting to control the, my…my time, my money…use my substances—the drugs, the alcohol—whenever I wanted when I decided. And my partner didn't think so. My partner took a certain care of me, and she'd say no, she wouldn't accept it, didn't accept it. And so we had arguments, right?* (Roni, cocaine, crack, SP).

*Yeah. I'd been around someone's house, and I'd been round there a few hours. And when I got home, straightaway, she was on my case. ‘Where you been? Where you been? What? You've been round there pissing it up have you?’ And it was, like, ‘Will you just shut up and leave me alone,’ you know*. (Roger, hazardous drinking, England).

The story Roger tells ended with a slap that is justified as the only way to ‘shut up’ his ‘nagging’ wife. There are strong parallels between Roger's account of his reported response to his wife's ‘nagging’ behaviour and the *respect* that Junior and Márcio—Brazilian participants—requires from a partner:

*I think there needs to be a bit of respect! Ask first where you were, how your day was. But no! Most of the time the woman doesn't even ask how her husband's day was, she only wants to know what…what really interests her—where you were and who you were with; and if you spent money, if you brought money for her to go to the market the next day*. (Júnior, cocaine, crack, SP).

*Women, like, any blip they get really worked up, you know? Women also don't take any crap, they don't know how to approach you and talk nicely. She comes by and already starts saying, ‘This drunkard’, and so on, because you drink and stuff. And alcohol makes you angry, you know? Then anything goes, it's where the conflicts and troubles start. I think violence is more present when you're involved in drinking, alcohol and stuff. And then there are the drugs*. (Márcio, alcohol, SP).

Júnior explains how a respectful wife should conduct herself—‘ask first where you were, how your day was’. Márcio states that women do not know how ‘to talk nicely’ to their intoxicated partners. Júnior recasts a woman's concern that there is money to buy food the next day as female acquisitiveness. In this meaning‐making, female anger and opposition to male substance use provokes IPV, which is enabled by intoxication, because as Márcio states ‘alcohol makes you angry’. While in these accounts disputes centre on male substance use, men also described IPV associated with drug sharing:

IV: *We'd just been and scored. We'd been out for the evening, all day, we'd just gone and got our heroin and was cooking it up and she was convinced that I'd had more of it and it just started and she was just getting in my face and I just lashed out*.

I: *In what way did you lash out?*

IV: *I just grabbed her round the throat and told her to fuck off and pushed her away*. (Jason, treatment for heroin, England).

Gilbert *et al*. 46 describe an expectation amongst drug using women that drugs will not be shared equally by their male partner. Jason presents perpetrating IPV in response to provocation from his partner (‘she was just getting in my face’) and craving to use, rather than an attempt on his part to assert male authority or entitlement. We are reminded that these accounts are ‘storied constructions of reality’ 17 and that an interview with Jason's partner might have constituted these events differently.

Other accounts made gender more salient. Men frequently described themselves as rendered inarticulate by a barrage of angry female criticism 47. Kevin describes physical violence as the only possible course of action in the face of verbal *bombardment*:

*It was always, yeah, an argument, yeah, where she'd be, like, the aggressor, like. But, you know, what I say is, a woman, yeah, she hasn't got nothing physical. She's just got it up there mentally. And once she starts going, I just can't keep up, and I think men can't keep up, you know, like, with that side of it, you know. So, a man will just, like, his defence is to lash out, whereas a woman wouldn't lash out, but she'd lash out with her tongue and, you know, it would just bombard you and bombard you, and in the end you'd be, like, ‘Shut up,’ you know, and you just can't take no more, you know*. (Kevin, treatment for heroin, crack, England).

Here, Kevin describes sets of male and female characteristics which normalise and make his violence legitimate. Presenting himself as the victim (‘she'd be, like, the aggressor’), Kevin uses the second person pronoun to suggest that any man would be able to ‘take no more’. The idea of a woman ‘lashing out with her tongue’ references an enduring discourse that derogates women's purported relative articulacy as nagging and controlling 48. This dynamic of a man rendered silent and hurt by what were described as ‘verbal attacks’ was presented as the context in which violence could escalate.

Looking back, either with the hindsight of more controlled substance use or abstinence, some men stated they understood their wife's anger:

*It was New Year's Eve, yeah because New Year's Eve we go to our relatives’, get all the kids together, the family together. But I abandoned my family and went to the streets, right? And then I'd arrive after the party. And that was when we had a lot of fights, arguments… I don't even blame her for it because I'm wrong, you know?* (Júnior, cocaine, crack cocaine, SP).

I: *So she was, sort of, angry?*

IV: *Hmm, very angry. Like, you know, like, I'd used her for years. All it was, was a bed and a bunk, you know, it was a bed. That's how she was feeling*. (Roger, abstinent from heroin and alcohol, England).

Feminist scholarship has consistently found that gender arrangements are reinforced in language and practice 49, 50, rely upon taken for granted, ‘hegemonic’ typifications of both masculinity and femininity 51 and are interactionally produced 52. In many of the accounts cited, the possibility of understanding violence as provoked relies upon sets of proscribed gender arrangements. These include scenarios where a substance using male partner fails to provide for the household or misuses limited resources, where substance use removes a man from family life and where a female partner challenges this behaviour. Such conflicts may thus revolve around men's failure to achieve ideal models of masculinity 53, 54 and women's refusal to fulfil cultural stereotypes of good womanhood which Jewkes has argued are amongst the most important variables for risk of IPV 55, 56

### Direct effects of intoxication

As found by previous research 20, 21, 22, 24 participants in our study presented intoxication as enabling *uncharacteristic* violence: in these narratives intoxication effected a transformation from their ‘real selves’ to different, aggressive, persons.

*Yeah. It's definitely, yes. Alcohol, it brings out the worst in me, you know. You know, when you're not drunk, you're calm and you can just ignore someone or walk out. But when you're drunk, something flicks a switch and you become violent*. (Wayne, treatment for heroin, hazardous drinking, England).

*If the person doesn't drink they are one person, if they drink they are another. Drinking, using other substances, they're another person. Even today I notice this! In the hostel, I see people that when they don't drink they're great, but when they drink*… (Augusto, alcohol, SP).

*And yeah I'd been on cocaine for a couple of days, I hadn't slept, been drinking which, yeah I saw him and I cut his face. That's not the only time I'd been done for violence, whenever I'd been done for violence I've always been intoxicated on alcohol or drugs*. (Craig, abstinent from alcohol and drugs, England).

Cultural expectancy of the link between intoxication and violence—that alcohol (and cocaine) promote aggression and violence because people expect it to—is acknowledged in research as a risk factor for IPV 44, 57. Peralta *et al*. suggest that the cultural association with alcohol and violence is not only gendered but linked to stage of life: in interviews, American college students associated *young* men with alcohol intoxication and violence in a type of ‘youthful masculinity’ 58. Our research participants' narratives of loss of control as a result of intoxication represent a competing account of IPV than that of provocation arising from gender violation or mutual conflict. Loss of control conflicts with idealised notions of mature masculinity. This conflict may be at the centre of men's presentation of intoxicated IPV as *uncharacteristic*. In contrast, men described violent loss of control as characteristic of female intoxicated behaviour however:

*When she drank, oh god, she would just smash up the place we are in, she'd come and attack me, try to stab me, you know?* (Adam, treatment for heroin, England).

*She did! She did that to me. She sent me running! Joana… Joana is a sad case! She has already pulled a knife on me, a chopper, a machete… Sad that little woman!* (Gil, hazardous drinking, cocaine, SP).

In both Brazilian and English men's accounts, alcohol and cocaine intoxication make differential cultural sense of IPV perpetrated by men and women. While male intoxicated loss of control represents loss of a true self, female intoxicated IPV is consistent with a view of female behaviour as more generally out of control.

### Respect, sexual jealousy and violent control of women

As suggested above, Brazilian research participants in particular described clear limits of acceptable behaviour for respectful women:

I: *Do you think she shouldn't go out alone?*

IV: *No*.

I: *Why do you think that?*

IV: *If she is respectful she shouldn't go out alone, no way, and she shouldn't walk around in mini‐shorts either in the middle of the street with her boobs exposed*. (Gil, hazardous drinking, cocaine, SP).

*Sometimes I'd come home from work, I'd go by the pub's entrance and she was sitting at the bar! She was with two other guys and three other women, drinking, snorting, you know? I used to look and say, ‘Shoot, this will not work!’ The woman, she gets home from work, gets things in order around the house and stuff, starts getting things ready, 'cause I am coming home!* (Jadson, cocaine/crack, SP).

The concept of *respect* is revealed as a gendered relation that circumscribes women's behaviour, dress and the leisure spaces they can inhabit. Sexual jealousy has been described as a key explanation for IPV perpetration amongst UK criminal justice populations 59. Amongst some of the Brazilian cohort, sexual betrayal—and this research participant suggests, catching one's partner *in flagrante delicto*, also provided warrant for IPV perpetration:

*She only gets beaten if she betrays him and he sees it. He sees it, no‐one comes to tell him, he catches her. Then I think there is aggression … we'll have aggressive reactions. Maybe you'll arrive there and you'll catch that act, maybe you'll even… attack to an extent that you'll kill someone* (Roni, alcohol, cocaine, crack cocaine, SP).

Roni here refers to a principle that if a man discovers his partner is betraying him, he will beat her; his use of the collective ‘we'll have an aggressive reaction’ removes himself from any specific offence and emphasises that this is a general principle rather than an individual response.

Very few men in the English cohort described having perpetrating severe attacks that hospitalised their partners, or having been immediately remanded in custody for a physical attack on their partner. It is instructive to note therefore how the more extreme acts of violence were reported in these interview accounts. Sexual jealousy as a result of a perceived betrayal was a rationale that in some cases provided sufficient explanations for IPV without recourse to explanations of substance use, competing for drugs or intoxication. Ralph, who reported having served custodial sentences for violent attacks on a series of partners, described IPV provoked by the perception of being publicly humiliated by his partner:

*We went into another pub in Road and she was all over this man, all over him, touching him up, and he was saying to her ‘If you were mine you, if you were my bird you'd be getting beaten up right now. You're being disrespectful’ Yes? Because me and him are there and she's in between stroking his hand, saying how lovely he is and all this sort of crap. ‘I mean, you know what, you do what you want to. I don't care’. So after a little bit I've had enough, finish my beer and I start walking down the road and she follows me again. So I go into a park and I just give her what she wants*. (Ralph, treatment for heroin).

Although these events are reported to take place in the context of an evening of drinking, the violent attack Ralph subsequently described—and what he is referring to as ‘what she wants’—is not *accounted for* by intoxication. Instead Ralph presents his actions as highly controlled and explains his violent attack in response to a perceived act of sexual betrayal. Ralph describes an alliance between himself and the man in the pub who is not only the object of his girlfriend's reported flirtation but provides moral endorsement for Ralph's violence. Ralph presents IPV as required to save face 60. Whether or not these events took place as described, we can note that betrayal alone *may* constitute sufficient rationale for IPV perpetration.

## Discussion

This research set out to address a gap in existing research through exploring the accounts of men who use substances and perpetrate IPV to elucidate the cultural conceptions that inform their violence. We distinguished three types of narrative: (i) disputes, centred on substance use, that escalate to IPV perpetration; (ii) IPV perpetration that is explained by uncharacteristic loss of control, as a result of intoxication; and (iii) IPV perpetration provoked by a perceived betrayal, in which substance use is incidental. In all types of accounts, hegemonic principles of male and female roles and behaviour provided a context for and make IPV perpetration explicable. Gender norms were revealed as a spectrum at the extreme end of whose breach, IPV perpetration was warrantable and deserved. Within this meaning‐making, substance misuse was more or less important as an explanation or excuse for IPV perpetration.

A strength of this study was the opportunity it afforded to compare the accounts of two culturally distinct groups of men, enabling the identification of commonalities and distinctions across the cohort. That we had a community rather than criminal justice sample made possible the analysis of accounts of everyday and sometimes mutual IPV perpetration that may be characteristic of the substance misusing population. We have described the protocol followed in terms of disclosure and limits to confidentiality. We are aware that this may have limited men's openness. We acknowledge that social desirability and underreporting are a limitation in all IPV research. Although we acknowledge that the gender and ethnicity of the interviewers may have influenced participants' disclosures 61; researchers were mindful of this throughout their analysis 62 and did not detect any trends in this respect. As DiCicco‐Bloom and Crabtree have noted 63, the interviewers' skill in developing rapport with the research participants is key to the generation of meaningful interview data.

Although men may use substance use as a means to excuse and avoid individual responsibility 64 our analysis suggest that accounts of a substance using life style and/or intoxication work differentially in narratives of IPV perpetration. In their review of neutralisation theory, Maruna and Copes 65 suggest that ‘excuses’ that separate past offending behaviour from the individual's ‘core’ or real self may be more associated with desistance from crime, than narratives that rely upon stable and generalisable attributions. IPV perpetration is distinct from other types of offences in its gendered dimension and, as discussed, its cultural explicability, if not acceptability. The generalising principles of *respect* evinced in some accounts, and where men account for IPV perpetration as a response to female betrayal, provide the sorts of stable attributions that Maruna and Copes suggest are linked to ongoing offending. These findings require further empirical and especially longitudinal investigation. While we have adopted a mainly thematic approach to IPV narratives, a more systematic analysis of the construction of IPV in treatment populations using discourse or conversation analysis 66 may furnish further insights.

We have been concerned to show how the accounts reveal cultural constructions of IPV in the two cohorts. Understanding this across countries is important in order to appreciate what might be generalisable and what might need to be culturally nuanced. Understanding the meaning‐making of substance using IPV perpetrators men is important in that it has implications for the treatment of both substance abuse and IPV. The inextricable relations between the two need to be more formally acknowledged and directly oriented to in treatment programs. To treat one or the other is to miss a significant aspect of the behaviour of many in these two populations. Substance use staff require training and guidance in order to identify IPV perpetration in initial assessment and key working. The differential character of accounts of IPV perpetration suggests the need for differential responses and referral routes. Further research needs to address whether a dual focus for those men with substance using lifestyles and IPV enhances outcomes.
